# Supplementary material for: Representations in human primary visual cortex drift over time
Source: Nat Commun. 2023 Jul 21;14:4422. doi: 10.1038/s41467-023-40144-w (PMC10361968; doi:10.1038/s41467-023-40144-w)
Supplement: Supplementary file 1 — Supplementary Information [file 41467_2023_40144_MOESM1_ESM.pdf]

Supplementary Information for:

Representations in human primary visual cortex drift over time

Zvi N. Roth* & Elisha P. Merriam

Laboratory of Brain and Cognition
National Institute of Mental Health, NIH,
Bethesda, MD, USA

*Corresponding author: zvi.roth@nih.gov

This file includes Figures S1 to S10

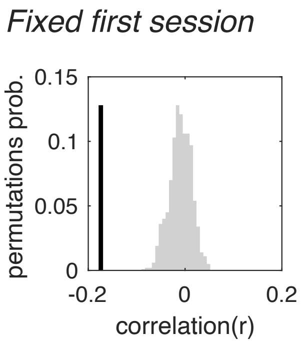

Fig S1. *Null distribution of correlation between* cvR^2 *and* Δ*session, while keeping the first session fixed in all permutations. Drift is still significant (p<0.001), indicating that it is not driven by different responses in the first session.*

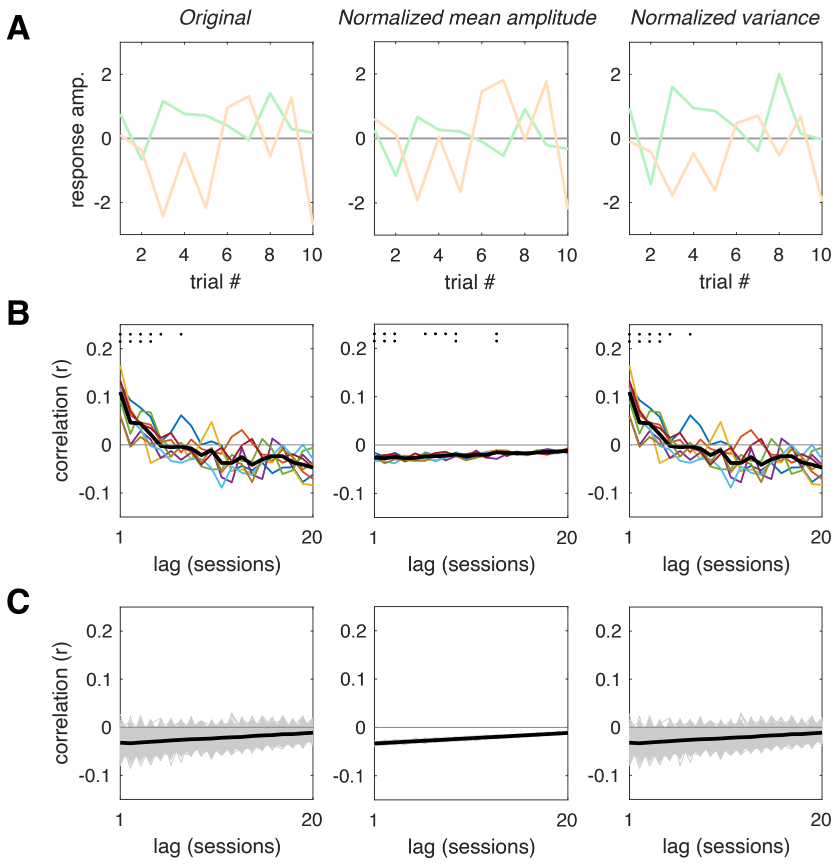

Fig S2. *Mean response amplitude gradually changes across sessions.* ***A****. Schematic illustration of original responses (left), responses after removing mean (center), and responses after normalizing variability (right).* ***B.*** *Autocorrelation of voxelwise mean beta, black dots indicate values significantly above zero (1 dot, p<0.05; 2 dots, p<0.01; uncorrected for multiple comparisons). Autocorrelation values are significantly above zero, up to a lag of 7 sessions, but only for original responses (left), and after normalized STD (right), but not after removing the mean response amplitude (center).* ***C.*** *Null distribution of autocorrelation values. Black line, mean across 1000 permutations; gray lines, values for individual permutations. Source data are provided as a Source Data file.*

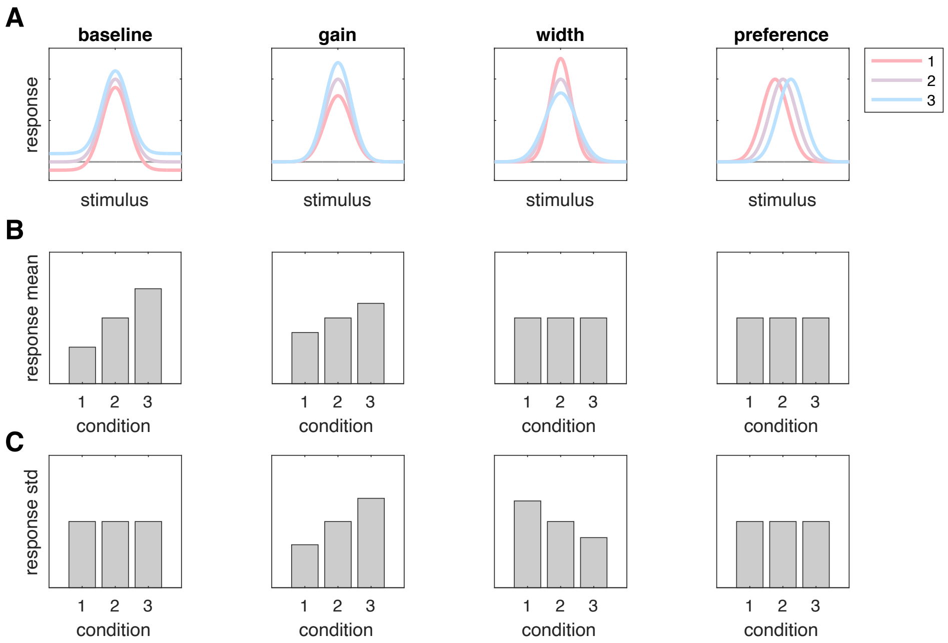

Fig S3. *Hypothetical changes in voxel tuning underlying drift.* ***A.*** *From left to right, possible changes are changes in: baseline, gain, tuning width, and stimulus preference. Three colored tuning curves are illustrated with different values for each possible tuning change.* ***B.*** *Mean response amplitude for each of the possible tuning curves. Changes in baseline and gain affect the mean, while changes in tuning width and preference do not.* ***C.*** *STD of response amplitudes for each of the possible tuning curves. Changes in gain and tuning width affect response STD, while changes in baseline and preference do not. These simulations suggest that only an additive baseline shift would affect the mean response amplitude, without a concomitant change in the variance of responses, consistent with the drift that we observed. Note that normalizing the mean amplitude cannot compensate completely for changes in the baseline, since each session included a different set of stimuli, and therefore was likely to evoke a different mean amplitude even if the baseline remained the same.*

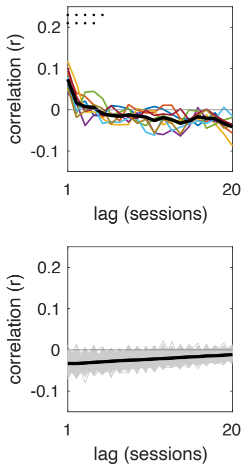

Fig S4. *The weight of the model's constant component changes gradually across sessions. Top: autocorrelation of the constant component. Black dots indicate values significantly above zero (1 dot, p<0.05; 2 dots, p<0.01; uncorrected for multiple comparisons). Bottom: Null distribution of constant weight autocorrelation values. These results confirm that, similar to response amplitudes of individual voxels, baseline response values as captured by the image-computable model gradually accumulate changes across sessions and are not simply fluctuating around a fixed mean. Source data are provided as a Source Data file.*

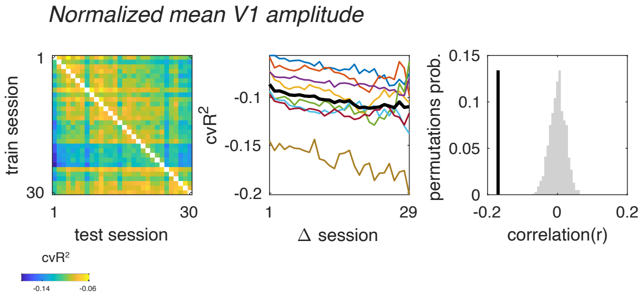

Fig S5. *Cross-session generalization after subtracting each session's mean response amplitude (i.e. mean beta) across the entire V1. Left, goodness-of-fit matrix. Center, Mean cvR^2 as function of number of intervening sessions between train and test sessions. Model predictive power decreases with time, indicating representational drift (r=-0.17, p<0.001). gray lines, individual subjects; thick black line, mean across subjects. Right, Black vertical line, empirical correlation between goodness-of-fit and number of intervening sessions. Gray histogram, null distribution of correlation values. Source data are provided as a Source Data file.*

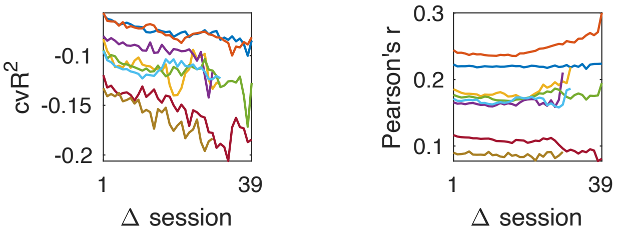

Fig S6. *Analysis using all sessions for each subject. cvR*^{2} *(left) and Pearson's r (right) as function of number of intervening sessions between training and test sessions. Source data are provided as a Source Data file.*

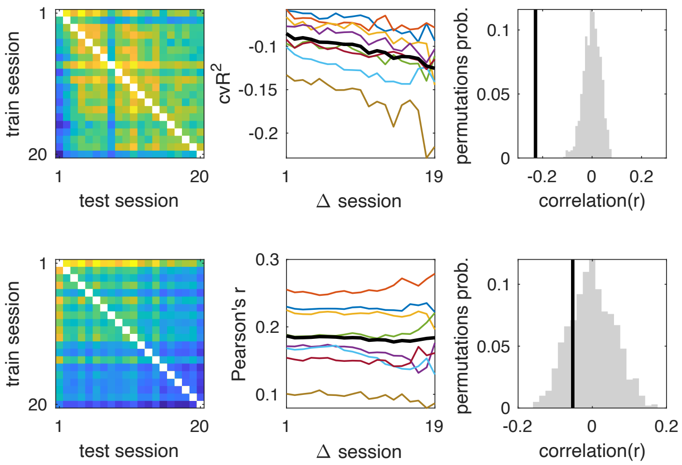

Figure S7. *Analysis limited to first 20 sessions. Top, cvR^2; Bottom, Pearson's r. Left: goodness-of-fit matrix. Center: goodness-of-fit as function of number of intervening sessions between training and test sessions. Colored thin lines, individual subjects; thick black line, mean across subjects. Right: null distribution of correlation values (gray histogram), and the empirical value (black vertical line). Source data are provided as a Source Data file.*

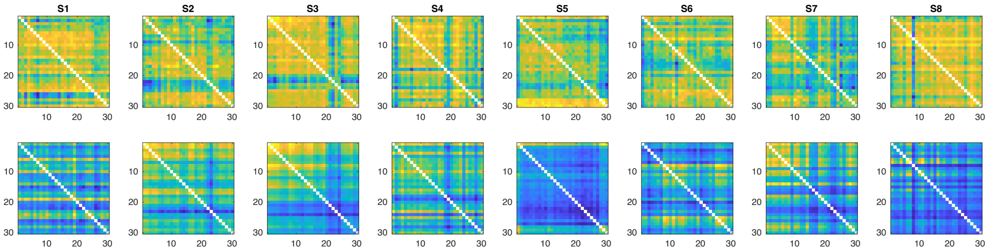

Figure S8. *cvR^2 (top) and correlation (bottom) matrices for individual subjects*

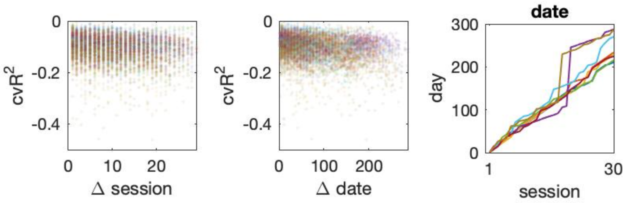

Figure S9. *Session number corresponds closely to session date. Left: cvR^2 as function of the number of intervening sessions between training and test sessions (delta_session). Each point represents a pair of training and testing sessions for a single subject. Center: cvR^2 as function of the number of intervening days between training and test sessions. Right: session day as a function of session number. Color in all panels reflects subject identity, as in Fig 1D.*

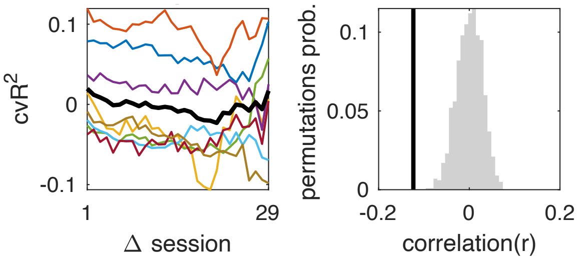

Figure S10. *Analysis limited to voxels with pRF R^2>0.75. Left: cvR^2 as function of number of intervening sessions between training and test sessions. Colored thin lines, individual subjects; thick black line, mean across subjects. Right: null distribution of correlation values (gray histogram), and the empirical value (black vertical line). Source data are provided as a Source Data file.*
