# Supplementary material for: Representations in human primary visual cortex drift over time
Source: Nat Commun. 2023 Jul 21;14:4422. doi: 10.1038/s41467-023-40144-w (PMC10361968; doi:10.1038/s41467-023-40144-w)
Supplement: Supplementary file 3 — Reporting Summary [file 41467_2023_40144_MOESM3_ESM.pdf]

nature portfolio

Corresponding author(s): Zvi N Roth

Last updated by author(s): Jun 18, 2023

Reporting Summary

Nature Portfolio wishes to improve the reproducibility of the work that we publish. This form provides structure for consistency and transparency in reporting. For further information on Nature Portfolio policies, see our Editorial Policies and the Editorial Policy Checklist.

Statistics

For all statistical analyses, confirm that the following items are present in the figure legend, table legend, main text, or Methods section.

n/a	Confirmed	
☐	☒	The exact sample size (*n*) for each experimental group/condition, given as a discrete number and unit of measurement
☐	☒	A statement on whether measurements were taken from distinct samples or whether the same sample was measured repeatedly
☐	☒	The statistical test(s) used AND whether they are one- or two-sided<br>*Only common tests should be described solely by name; describe more complex techniques in the Methods section.*
☒	☐	A description of all covariates tested
☒	☐	A description of any assumptions or corrections, such as tests of normality and adjustment for multiple comparisons
☐	☒	A full description of the statistical parameters including central tendency (e.g. means) or other basic estimates (e.g. regression coefficient) AND variation (e.g. standard deviation) or associated estimates of uncertainty (e.g. confidence intervals)
☐	☒	For null hypothesis testing, the test statistic (e.g. F, t, r) with confidence intervals, effect sizes, degrees of freedom and P value noted<br>*Give P values as exact values whenever suitable.*
☒	☐	For Bayesian analysis, information on the choice of priors and Markov chain Monte Carlo settings
☒	☐	For hierarchical and complex designs, identification of the appropriate level for tests and full reporting of outcomes
☐	☒	Estimates of effect sizes (e.g. Cohen's *d*, Pearson's *r*), indicating how they were calculated

Our web collection on statistics for biologists contains articles on many of the points above.

Software and code

Policy information about availability of computer code

Data collection	From Allen et al. (2021) describing data collection for the NSD: Psychophysics Toolbox 3.0.14, MATLAB R2018a, Meadows web-based platform (http://meadows-research.com). Images used for NSD were taken from the Common Objects in Context database (Lin et al., 2014) (https://cocodataset.org).
Data analysis	MATLAB R2021 (https://www.mathworks.com/products/matlab.html), mrTools v4.7 (https://github.com/justingardner/mrTools), Steerable Pyramid (https://github.com/LabForComputationalVision/matlabPyrTools), FreeSurfer v6 (https://surfer.nmr.mgh.harvard.edu/), Custom MATLAB code (https://github.com/elimerriam/repDriftNSD/).

For manuscripts utilizing custom algorithms or software that are central to the research but not yet described in published literature, software must be made available to editors and reviewers. We strongly encourage code deposition in a community repository (e.g. GitHub). See the Nature Portfolio guidelines for submitting code & software for further information.

Data

Policy information about availability of data

All manuscripts must include a data availability statement. This statement should provide the following information, where applicable:

- Accession codes, unique identifiers, or web links for publicly available datasets
- A description of any restrictions on data availability
- For clinical datasets or third party data, please ensure that the statement adheres to our policy

The NSD dataset is freely available at http://naturalscenesdataset.org. Images used for NSD were taken from the Common Objects in Context database (Lin et al., 2014) (https://cocodataset.org). Source data are provided with this paper.

Research involving human participants, their data, or biological material

Policy information about studies with human participants or human data. See also policy information about sex, gender (identity/presentation), and sexual orientation and race, ethnicity and racism.

Reporting on sex and gender	The NSD data consists of 2 males and 6 females. We do not know which of the 8 participants were the 2 males.
Reporting on race, ethnicity, or other socially relevant groupings	We have no information on race, ethnicity, or other socially relevant groupings of the NSD participants.
Population characteristics	All participants were healthy young adults between 19–32 years old, and all provided informed written consent.
Recruitment	Participants were recruited through advertisements to the local community and were screened based on ability to participate in a long-term neuroimaging study. In addition, participants were selected based on data quality from an initial 7T fMRI session.
Ethics oversight	This study was approved by the Institutional Review Board at National Institutes of Health (protocol number 93-M-0170). The NSD study was approved by the University of Minnesota Institutional Review Board

Note that full information on the approval of the study protocol must also be provided in the manuscript.

Field-specific reporting

Please select the one below that is the best fit for your research. If you are not sure, read the appropriate sections before making your selection.

☒ Life sciences ☐ Behavioural & social sciences ☐ Ecological, evolutionary & environmental sciences

For a reference copy of the document with all sections, see nature.com/documents/nr-reporting-summary-flat.pdf

Life sciences study design

All studies must disclose on these points even when the disclosure is negative.

Sample size	8 subjects are included in the NSD. We did not choose the sample size, and analyzed all participants' data.
Data exclusions	We excluded any sessions beyond session 30, since some participants had only 30 sessions.
Replication	We did not attempt to replicate our findings.
Randomization	For permutation tests the order of sessions was randomized.
Blinding	Blinding is not relevant to this study since the participants were not divided into groups.

Reporting for specific materials, systems and methods

We require information from authors about some types of materials, experimental systems and methods used in many studies. Here, indicate whether each material, system or method listed is relevant to your study. If you are not sure if a list item applies to your research, read the appropriate section before selecting a response.

Materials & experimental systems

n/a	Involved in the study
☒	☐ Antibodies
☒	☐ Eukaryotic cell lines
☒	☐ Palaeontology and archaeology
☒	☐ Animals and other organisms
☒	☐ Clinical data
☒	☐ Dual use research of concern
☒	☐ Plants

Methods

n/a	Involved in the study
☒	☐ ChIP-seq
☒	☐ Flow cytometry
☒	☐ MRI-based neuroimaging
